# Physicochemical properties, anticancer and antimicrobial activities of metallic nanoparticles green synthesized by *Aspergillus kambarensis*

**DOI:** 10.1049/nbt2.12070

**Published:** 2021-11-23

**Authors:** Mohammadhassan Gholami‐Shabani, Fattah Sotoodehnejadnematalahi, Masoomeh Shams‐Ghahfarokhi, Ali Eslamifar, Mehdi Razzaghi‐Abyaneh

**Affiliations:** ^1^ Department of Biology, Science and Research Branch Islamic Azad University Tehran Iran; ^2^ Department of Mycology Faculty of Medical Sciences Tarbiat Modares University Tehran Iran; ^3^ Department of Clinical Research Pasteur Institute of Iran Tehran Iran; ^4^ Department of Mycology Pasteur Institute of Iran Tehran Iran

**Keywords:** Anticancer activity, Antimicrobial activity, *Aspergillus kambarensis*, Green synthesis, *in vitro* cytotoxicity, Metallic nanoparticles

## Abstract

In the present study, metal and metal oxide nanoparticles were successfully synthesized using *Aspergillus kambarensis*. UV–Vis spectroscopy showed maximum absorbance of 417 nm for silver (AgNPs), 542 nm for gold (AuNPs), 582 nm for copper (CuNPs) and 367 nm for zinc oxide (ZnONPs) nanoparticles. Fourier transform infrared spectroscopy indicated the presence of various mycochemicals with diverse functional groups in the fungal cell‐free filtrate. Transmission electron microscopy revealed mono and poly dispersed particles with an estimate size of 50 nm and different shapes for synthesized manufacture metallic nanoparticles (MNPs. Dynamic light scattering confirmed that MNPs were dispersed in the size range less than 50 nm. Zeta potential analysis showed values of −41.32 mV (AgNPs), −41.26 mV (AuNPs), −34.74 mV (CuNPs) and 33.72 mV (ZnONPs). X‐ray diffraction analysis demonstrated crystalline nature for MNPs. All the synthesized MNPs except AuNPs showed strong antifungal and antibacterial activity in disc diffusion assay with growth inhibition zones of 13.1–44.2 mm as well as anticancer activity against HepG‐2 cancer cell line with IC_50_ in the range of 62.01–77.03 µg/ml. Taken together, the results show that biologically active MNPs synthesized by *A. kambarensis* for the first time could be considered as promising antimicrobial and anticancer agents for biomedical applications.

## 1 INTRODUCTION

Nanotechnology is an extensive and interdisciplinary scope of research which has the potential for revolutionizing the route in which materials and products are manufactured and the range and character of functionalities that can be accessed [1, 2]. Different physicochemical approaches have been broadly used to manufacture metallic nanoparticles (MNPs), in which the stability and the use of hazardous toxic chemicals is the significant topic of paramount concern [3]. It means that the use of toxic and hazardous chemicals on the manufacture of MNPs and non‐polar compounds in the manufacture procedure limits their applications in therapeutic fields [4]. Consequently, the progress of clean, eco‐friendly, biocompatible and non‐toxic techniques for MNPs production deserves merit [5].

Biological approaches could be considered as an alternative to the conventional methods for the manufacture of MNPs [6, 7]. Metal salts (silver, aluminium, copper, gold, zinc, iron, titanium, and palladium) have been widely used for the green bio‐manufacturing of MNPs [8, 9, 10, 11, 12, 13, 14, 15]. Some well‐known examples of microorganisms used for MNPs synthesis include bacteria, yeast, fungi and algae [5]. A wide array of fungi from different genera and species have been explored for MNPs mycosynthesis such as AgNPs, ZnONPs, AuNPs, Al_2_O_3_NPs, CdSNPs, CuNPs and tellurium NPs [16, 17]. Fungi are more advantageous as compared to other microorganisms in various ways [18]. They are easy to grow, handle and manufacture and fungal biomasses are more suitable compared to plant and bacteria resources for use in bioreactors [19]. Likewise, the extracellular secretions of reductive fungal enzymes and proteins can be easily used in downstream processing [20]. Likewise, since the MNPs are free from unnecessary cellular components as they participate outside the cell, they can be directly use in various applications without need for any further purification [9].

MNPs have proved to be most efficient as they have good antimicrobial activity against bacteria, fungi, and other microorganisms [21]. Nowadays, MNPs have gained attention in cancer research as diagnostic and therapeutic agents in treatment of cancer cells [22].

The present study reports for the first time the biogenic production of MNPs using *Aspergillus kambarensis* and the biological activity of synthesized NPs against cancer cell‐lines, as well as pathogenic fungi and bacteria.

## 2 MATERIALS AND METHODS

### 2.1 Microorganisms and chemicals

*A. kambarensis* PFCC 411‐132 was used for the mycobiosynthesis of MNPs. The fungus was obtained from Pathogenic Fungi Culture Collection of the Pasteur institute of Iran. All culture media and chemicals including potato dextrose broth (PDB; Merck), Sabouraud dextrose agar (SDA; Merck), copper (II) sulphate pentahydrate salt (CuSO_4_·5H_2_O, 99% purity, Sigma‐Aldrich), silver nitrate (AgNO_3_, 99% purity, Sigma‐Aldrich), hydrogen tetrachloroaurate (III) (HAuCl_4_·3H_2_O, 99% purity, Sigma‐Aldrich), and zinc sulphate (ZnSO_4_·7H_2_O, 99% purity, Sigma‐Aldrich) were of analytical grade purchased from international companies.

### 2.2 Preparation of fungal cell‐free extract

The mycelia extract of *A. kambarensis* was prepared according to Hu et al. [23] with some modifications. In brief, fungal spore suspension (10^6^ spores/ml) was inoculated in PDB medium (200 ml) in 500 ml Erlenmeyer flasks and incubated at 28°C for 96 h with continuous shaking at 200 rpm in a shake incubator (LABTECH). After incubation time, the residual components of the culture medium in the mycelia were removed by filtration and washed five times with deionized water. The harvested fungal mycelia (wet weight, 30–40 g) was transferred into 200 ml deionized water in 500 ml Erlenmeyer flasks and incubated at 28°C for 48 h with shaking at 200 rpm for cell‐free extraction. The fungal biomass was harvested using filtration through Whatman filter paper and the cell‐free filtrate was used in the next steps.

### 2.3 Synthesis and purification of MNPs

The extracellular mycobiosynthesis of the MNPs (Ag, Au, Cu and ZnO NPs) were carried out by standard methods [21, 22, 23, 24]. One millilitre of fungal cell‐free filtrate prepared above was mixed with 1 ml of 1 mM of each metal salts (CuSO_4_, AgNO_3_, HAuCl_4_ and ZnSO_4_) solutions. The mixtures were shaken for 96 h at dark conditions in room temperature. The secretion of bioactive substances into the cell‐free filtrate led to the production of MNPs. Cell‐free filtrate without metal salts and only metal salts (1 mM) were included in the experiment as positive and negative controls, respectively.

### 2.4 Physical characterization of MNPs

Purified MNPs were characterized using different instruments such as UV–visible spectroscopy, dynamic light scattering (DLS), Fourier transform infrared spectroscopy **(**FTIR), transmission electron microscopy (TEM) and X‐ray diffraction (XRD).

#### 2.4.1 UV–visible analysis

The successful mycobiosynthesis of MNPs were determined by measuring the absorbance of the MNPs solution at various time intervals (1–96 h) at 300–800 nm using UV–Vis spectrophotometer (Perkin Elmer EZ301). After the 96 h reaction, the mixture containing MNPs was centrifuged (20,000 rpm, room temperature, 20 min), the supernatant was removed, and washed (two times) using deionized water. Then, these MNPs were lyophilized and this lyophilized powder was used for further physical characterization.

#### 2.4.2 Zeta potential and DLS analysis

The zeta potential measurement was performed to estimate the surface charge of each mycosynthesized MNPs, which reflects their stability. The colloidal suspension of each MNPs was 10‐fold diluted with ultrapure water and homogenized using ultrasonic homogenizer for 30 min at 42 Hz. The MNPs solution was then filtered through a Millipore filter (0.22 μm) and analysed using Zetasizer (Malvern zetasizer nano ZEN3600). The size of the MNPs was assessed in terms of DLS using the above method and the data was plotted in graph [19].

#### 2.4.3 TEM analysis

MNPs were dispersed in deionized water. A small drop of thin dispersion is placed on a ‘staining pad/pigment pad’. Carbon‐coated copper grid was inserted into the drop with the coated side up. The films of MNPs on TEM grids were air dried and observed by a TEM‐Philips CM200 at an accelerating voltage of 80– 200 kV [25, 26].

#### 2.4.4 XRD analysis

The air dried MNPs were coated onto XRD grid and analysed for the formation of MNPs by Philips PW 1390 X‐Ray diffractometer at a voltage of 20–100 kV. Two gram of fine powders of each MNPs with the thickness around 0.2 cm with sample uniform layer on one side was used for taking diffractogram. The 2θ‐diffracted angles intensities were recorded and graphs were carried out using the standard method [25, 26].

#### 2.4.5 FTIR analysis

For FTIR (Shimadzu FTIR 8400S) analysis, 10 mg of each MNPs was used and the spectra were scanned (400–4000 cm^−1^, resolution of 4 cm^−1^) for the characterization of chemical functional groups present over the surface of mycosynthesized MNPs.

### 2.5 Assessment of antimicrobial activity of MNPs

#### 2.5.1 Microorganisms used

Pure cultures of bacterial and fungal strains used were *Escherichia coli* PTCC 1860, *Bacillus subtilis* PTCC 1254, *Proteus vulgaris* PTCC 1861, *Staphylococcus aureus* PTCC 1112, *Candida albicans* PTCC 5027, *Candida tropicalis* PTCC 5028, *Fusarium oxysporum* PTCC 5115 and *Aspergillus niger* PTCC 5154. Bacterial cultures were maintained on nutrient agar and fungal cultures were kept on SDA medium.

#### 2.5.2 Antimicrobial activity

The MNPs mycobiosynthesized from *A. kambarensis* cell‐free filtrate were tested for their antimicrobial activity by standard disc diffusion technique [27, 28]. Bacteria were sub‐cultured in nutrient broth (37°C, 200 rpm, 24 h). Fungi were grown in Sabouraud dextrose broth (SDB, 28°C, 200 rpm, 48 h). Cell suspension of all tested microorganisms was swabbed uniformly using sterile cotton swab on the individual Petri dish. Sterile blank discs of 6.0 mm diameter (HiMedia) containing 50 and 100 µg/ml of each MNPs were placed on each Petri dish. Streptomycin sulphate (for bacteria) and fluconazole (for fungi) standard discs were used in a similar manner. After incubation overnight at 37°C for 24 h (for bacteria) and 28°C for 48 h (for fungi), inhibition zones around disks were measured.

### 2.6 Assessment of anticancer activity of MNPs

#### 2.6.1 Cell‐line and cell culture

The HepG‐2 cell‐lines were maintained on DMEM medium with low glucose supplemented with 10% FBS, 60 mg/L penicillin, 100 mg/L streptomycin and 50 μl/L amphotericin B. The cells were cultured in a humid atmosphere (5% CO_2_ at 37°C).

#### 2.6.2 MTT assay of MNPs

The cytotoxic property of each MNPs on HepG‐2 cell‐line was evaluated using MTT (3‐[4,5‐dimethylthiazol‐2‐yl]‐2,5 diphenyl tetrazolium bromide) assay [29, 30]. The cells (1 × 10^5^ cells/well) were plated in a 96‐well plate. After 24 h, the medium was replaced with 100 µl of medium containing each MNPs at various concentrations (0–100 µg/ml) and incubated for 24 h. At the end of the treatment period, the cells were washed with sterile phosphate‐buffered saline (PBS; pH 7.2, two times), and MTT (0.5 mg/ml in PBS) was added to each well and incubated for 3 h at 37°C in CO_2_ incubator. MTT was then aspirated and the formazan crystals were dissolved in 100 μl of dimethyl sulfoxide. The formazan formed was measured using an ELISA reader (Organon Teknika) at 570 nm.

#### 2.6.3 Cell analysis by optical microscopy

Study of morphological changes of apoptotic HepG‐2 cells was performed according to previous methods with some modifications [31, 32]. Briefly, the cells (1 × 10^5^) were treated with each mycosynthesized MNPs at IC_50_ concentration on sterile glass cover slips and incubated for 24 h. The cover slips were lightly mounted on slides for analysing morphological changes. The morphological changes of the HepG‐2 cells were observed using phase contrast inverted optical microscopy (Olympus).

#### 2.6.4 Analysing cells by dual acridine orange/ethidium bromide

Study of acridine orange (AO)/ethidium bromide (EB) fluorescent staining was performed according to previous methods with some modifications [21, 22]. Briefly, the cells (1 × 10^5^) were deposited in the 24‐well microplate and treated by each mycosynthesized MNPs at IC_50_ concentrations (37°C for 24 h). The cells were collected, washed once with sterile PBS and then suspended with sterile PBS. For AO/EB fluorescent staining, 1 µl of AO/EB (Sigma) dye mixture (10 mg/ml of AO/EB in PBS) was added to cell suspension (9 µl) and then covered with a cover slip on a clean microscopic slide. After incubation for nearly 3 min, the cells were observed under a fluorescence microscope (Olympus) with appropriate excitation filter set at 510–590 nm.

#### 2.6.5 Analysing cells by Hoechst

Briefly, the cells (1 × 10^5^) were treated with each mycosynthesized MNPs at IC_50_ concentration in 96‐well microplates and incubated for 24 h at 37°C. The cells were washed twice with sterile PBS and incubated with Hoechst 33342 solution (50 µl, 100 µg/ml, Sigma) at room temperature in dark condition for 20 min [22, 32]. The cells were washed in sterile PBS, and then cell morphology was observed under inverted fluorescence microscope (Olympus).

#### 2.6.6 Analysing cells by rhodamine

Briefly, the cells (1 × 10^5^) were seeded in six‐well microplates and treated by each mycogenic MNPs with IC_50_ concentrations and incubated in 5% CO_2_, 24 h at 37°C. The cells were washed twice with sterile PBS and fixed with methanol and 4% formaldehyde for 20 min and kept at room temperature for drying. Then the cells were stained with Rhodamine‐123 (50 µl, 100 µg/ml, Sigma) for 10 min in dark conditions at room temperature. Then, cell morphology was observed under inverted fluorescence microscope (Olympus) [32].

#### 2.6.7 Analysing cells by DNA fragmentation

DNA fragmentation was performed according to Prasannaraj and Venkatachalam [32]. Cells (1 × 10^5^) were exposed to each mycogenic MNPs at IC_50_ concentrations and incubated in 5% CO_2_, 24 h at 37°C. The cells were lightly scraped and harvested using centrifugation. The cells were suspended in 10 ml of 10 mM TE buffer solution (pH 8.0, 10 mM EDTA, 10 mM Tris‐HCl, 2% SDS, and 20 mg/ml proteinase K). The mixture was incubated for 3 h at 37°C, followed by DNA extraction. The DNA was treated with DNase‐free RNase (20 mg/ml concentration, 4°C and 45 min) and precipitated with sodium acetate (100 ml, 2.5 M) and three volumes of ethanol. DNA fragmentation study was carried out with 10 µl of DNA prepared from treated cells by each mycosynthesized MNPs at IC_50_ concentration and analysed by electrophoresis on 1.5% agarose gel containing EB for a period of 45 min at 100 V.

#### 2.6.8 Statistical analysis

Statistical analysis was performed by graph pad prism programme. Each result is expressed as the mean ± standard deviation of two independent experiments in triplicate. *p* values less than 0.05 (ANOVA; *p* < 0.05) were considered significant.

## 3 RESULTS AND DISCUSSION

### 3.1 In vitro mycosynthesis of MNPs

In the present study, a simple and green approach for MNPs biosynthesis was designed using the filamentous fungus *A. kambarensis* that was tolerant to 1 mM of metal salts solution. When the fungal cell‐free filtrate was mixed with each AgNO_3_, CuSO_4_, HAuCl_4_, and ZnSO_4_ solution, the colour of the reaction mixture turned from white to dark brown (AgNPs), blue to green (CuNPs), yellow to purple (AuNPs) and yellow to colourless (ZnONPs) indicating successful nanoparticle synthesis. Cell‐free mycobiosynthesis has many advantages over the intracellular biosynthesis that involves additional steps for MNPs purification after biosynthesis [18, 19]; extracellular biosynthesis is inexpensive, favours large‐scale manufacture and requires simple downstream process for purification steps [3, 4, 5, 6, 7, 8]. The method used in the present study for MNP mycobiosynthesis did not need for any additional steps like ultrasound, chemicals or reaction with detergents to eliminate undesirable components. Therefore, only centrifugation was used for elimination of the residue that remains in the MNPs suspension, thus, this approach could be considered as suitable for large‐scale production of MNPs.

### 3.2 Characterization of mycosynthesized MNPs

#### 3.2.1 UV–visible analysis

**FIGURE 1:**
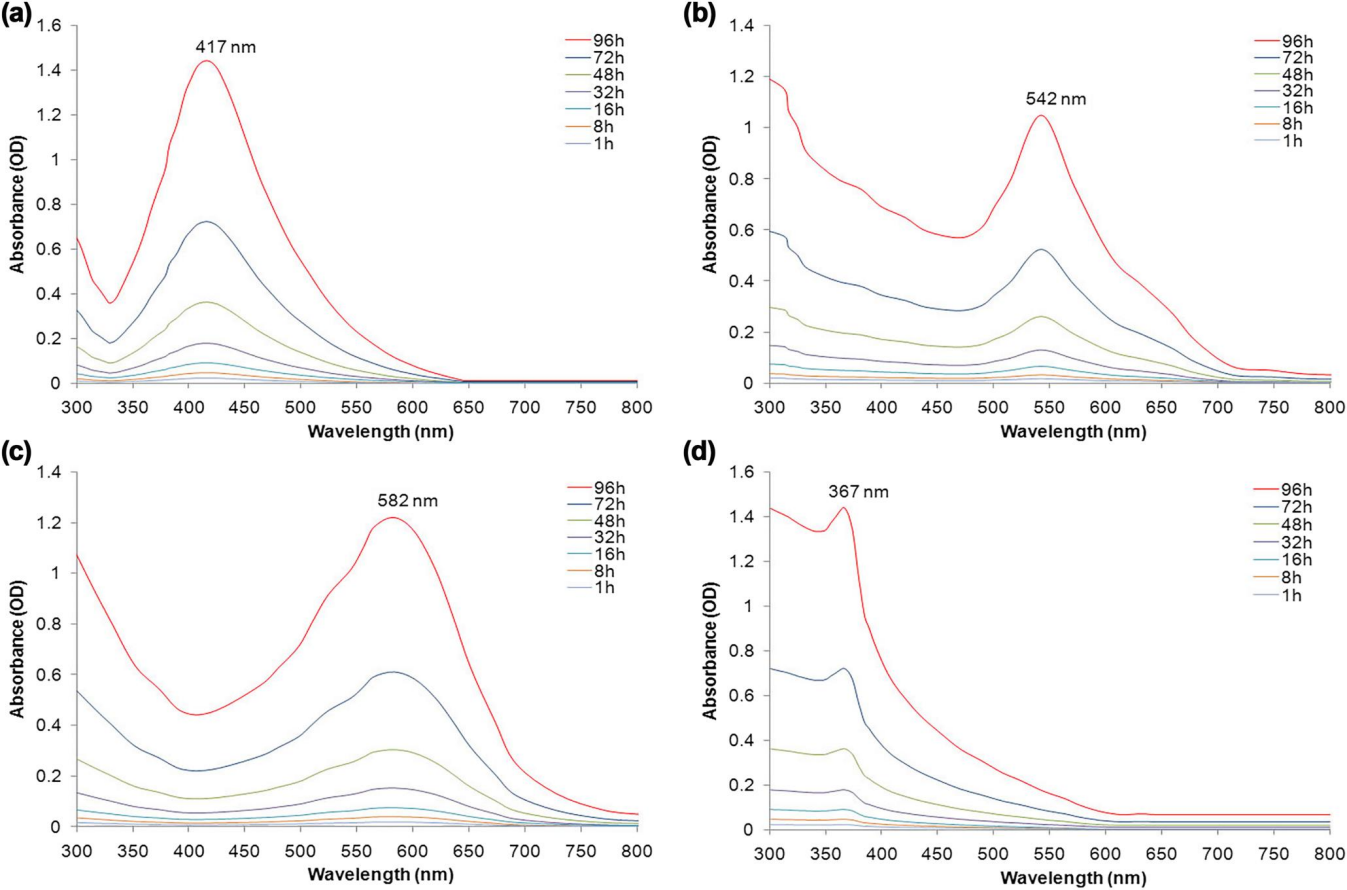
UV–Vis spectra recorded as a function of time of reaction of 1 mM, (a) AgNO_3_, (b) HAuCl_4_, (c) CuSO_4_ and (d) ZnSO_4_ with cell‐free filtrate of *Aspergillus kambarensis*. The results displayed the range of AgNPs (417 nm), AuNPs (542 nm), CuNPs (582 nm) and ZnONPs (367 nm)

The results of UV–visible spectroscopy analyses of MNPs are summarized in Figure 1. After 96 h of reaction, the maximum absorbance was observed in UV–vis analysis for AgNPs (417 nm, Figure 1a) AuNPs (542 nm, Figure 1b), CuNPs (582 nm, Figure 1c) and ZnONPs (367 nm, Figure 1d). In agreement with the result of this study, previous reports showed that peaks of UV absorption of Ag, Au, Cu and ZnO NPs were in the range from 380 to 450 nm, 520 to 580 nm, 580 to 590 nm, 340 to 380 nm, respectively [33, 34, 35, 36, 37].

#### 3.2.2 Zeta potential and DLS analysis

**FIGURE 2:**
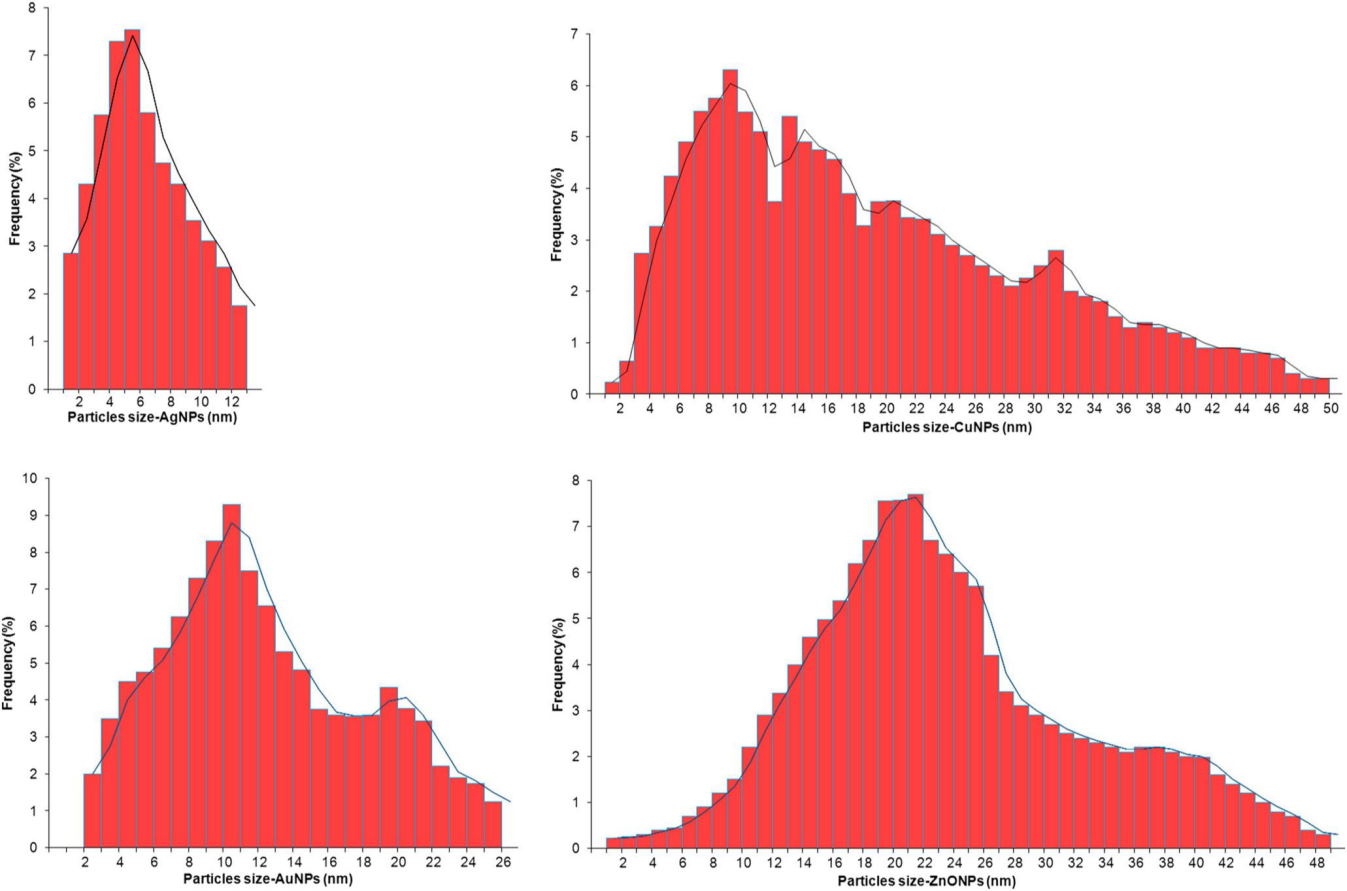
Hydrodynamic size of nanoparticles was measured using dynamic light scattering. The diameter of mycosynthesized AgNPs, AuNPs, CuNPs and ZnONPs were estimated less than 50 nm

**FIGURE 3:**
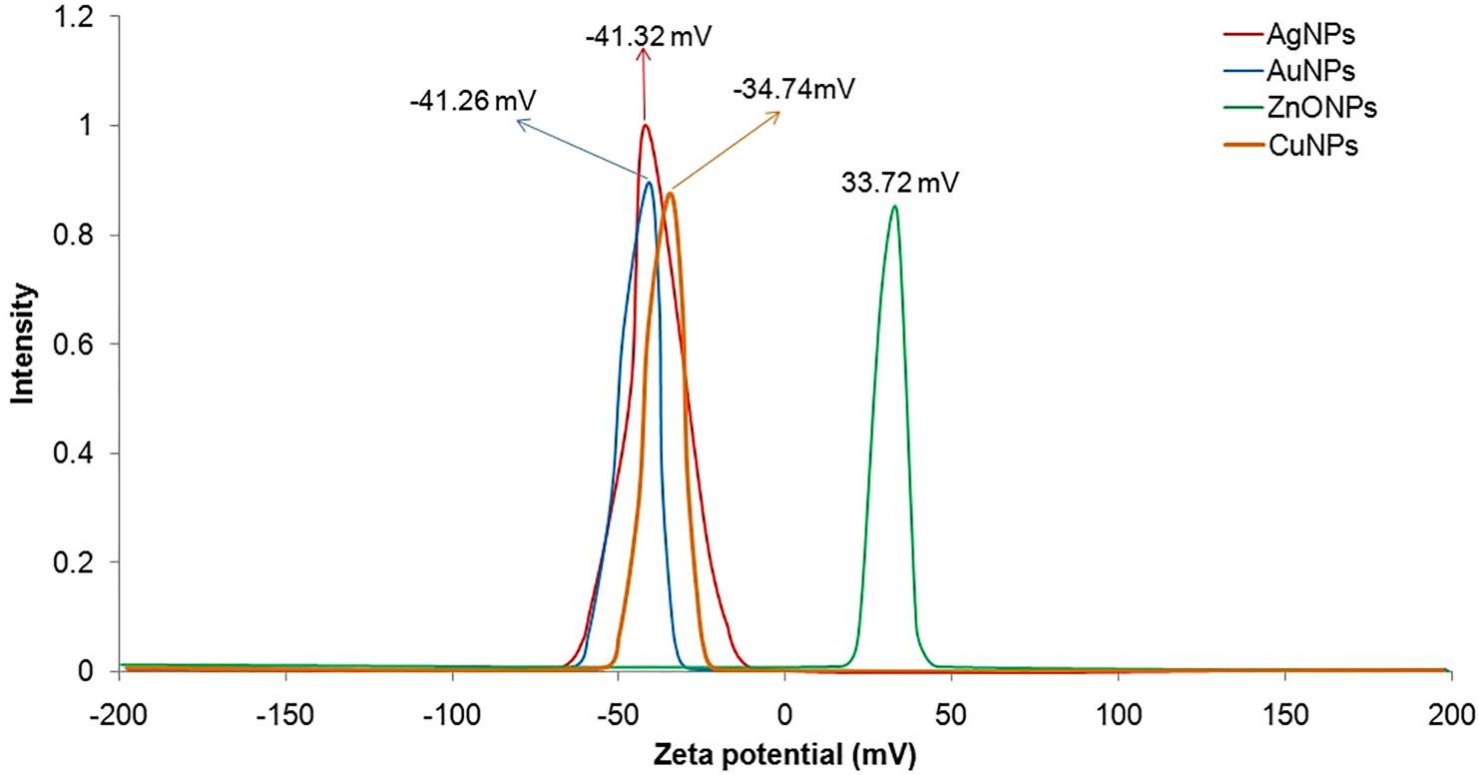
Zeta potential details of MNPs synthesized by cell‐free filtrate of *Aspergillus kambarensis*

DLS data displays that particles are dispersed with size range of less than 50 nm (Figure 2). The stability of MNPs was determined using zeta potential measurements. The zeta potential value is used as a key indicator of the particles’ stability dispersion. MNPs synthesized by cell‐free filtrate of *A. kambarensis* displayed a Zeta potential value of −41.32 mV (AgNPs), −41.26 mV (AuNPs), −34.74mV (CuNPs) and 33.72 mV (ZnONPs). Figure 3 shows that capped bio‐molecules are highly negatively/positively charged surfaces. The strong negative/positive charge on the bio‐fabricated MNPs give them colloidal stability as similar charges have electrostatic repulsion, thus preventing MNPs aggregation and providing long‐term stability. Indeed, solutions with zeta potential smaller than −30 mV or larger than +30 mV were considered as stable [34, 38].

#### 3.2.3 TEM analysis

**FIGURE 4:**
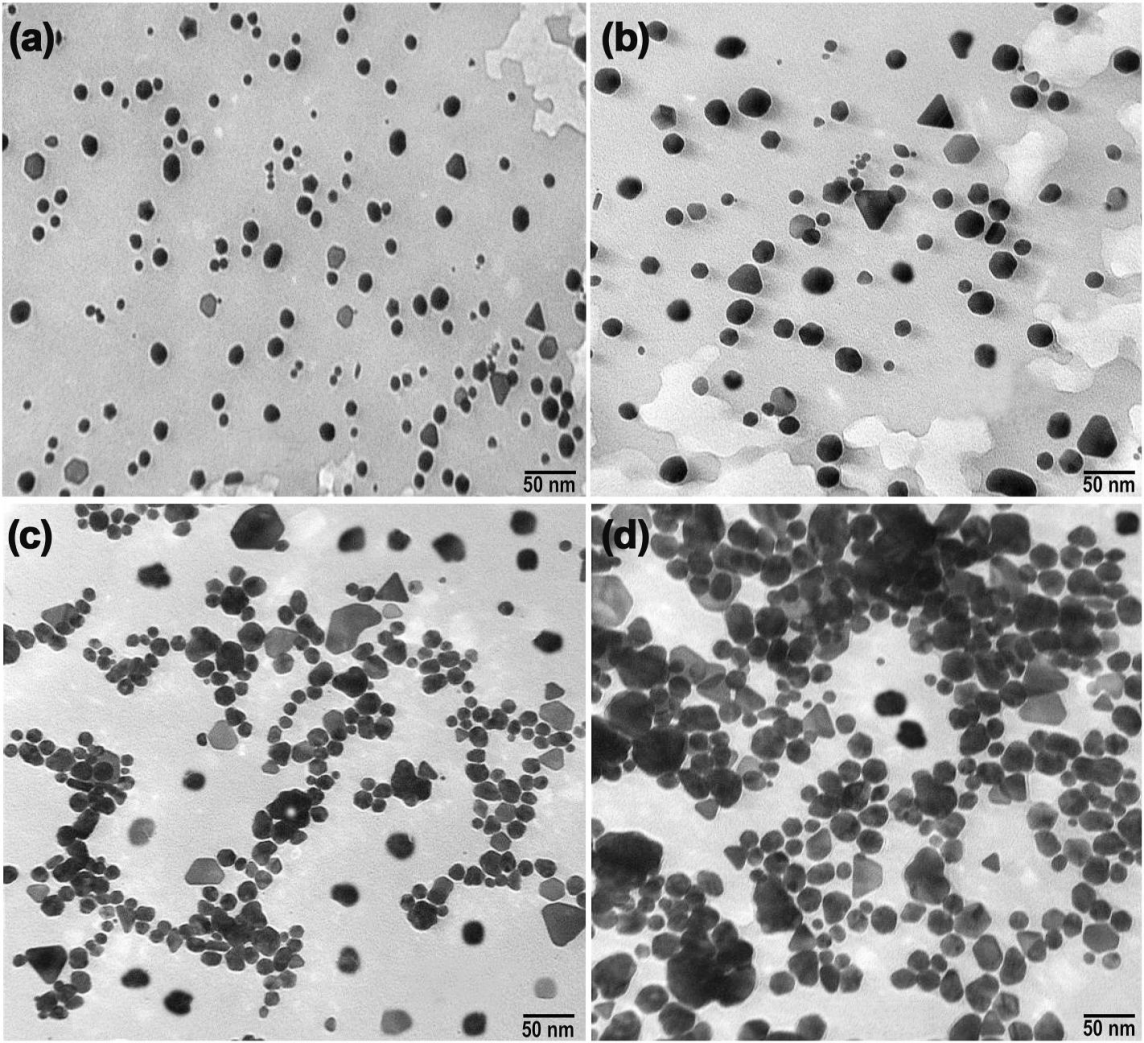
Transmission electron microscopy images of AgNPs (a), AuNPs (b), CuNPs (c) and ZnONPs (d) mycosynthesized by cell‐free filtrate of *Aspergillus kambarensis* show an estimate size of less than 50 nm for manufacture metallic nanoparticles

Figure 4 represents the TEM results for the Ag, Au, Cu and ZnO NPs synthesized by the fungal cell‐free filtrate. MNPs had a size less than 50 nm. The TEM micrograph was clearly revealed MNPs (Ag, Au, Cu and ZnO NPs) in different shapes. These properties are consistent with those reported for nanoparticle synthesis using a variety of fungi [33, 39, 40, 41].

#### 3.2.4 XRD analysis

**FIGURE 5:**
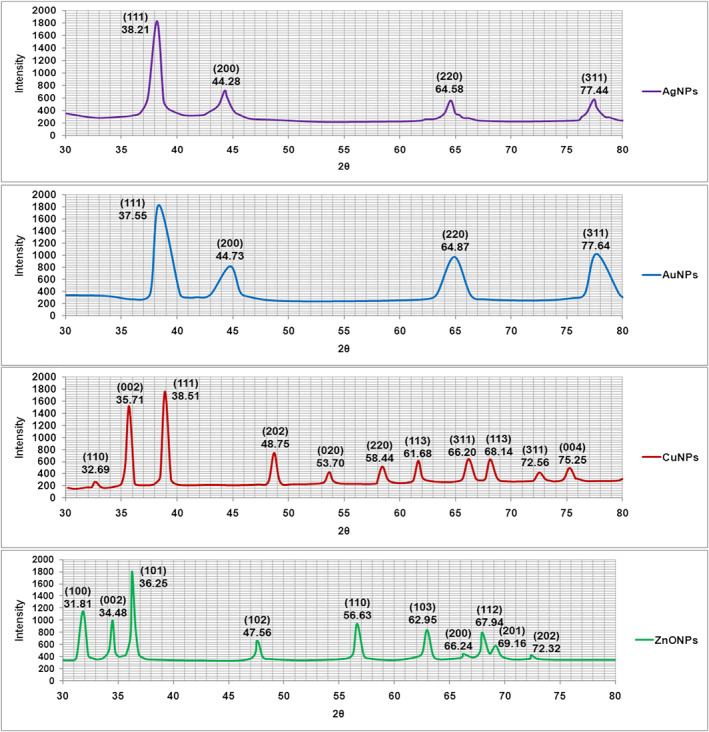
X‐ray diffraction (XRD) pattern recorded from the freeze‐dried powder of the manufacture metallic nanoparticles reaction mixture. The obtained XRD spectrum confirmed that the mycosynthesized Ag, Au, Cu and ZnONPs were in nanocrystal form and crystalline in nature

The results of XRD analysis of MNPs are shown in Figure 5. XRD pattern clearly predicted that MNPs were formed by bio‐reduction of cell‐free extract of *A. kambarensis*. For AgNPs, the characteristic diffraction peaks at 2θ values were predicted at 38.21, 44.28, 64.58 and 77.44, respectively. When compared with the standard (JCPDS card number 04‐0783), the obtained XRD spectrum confirmed that the mycosynthesized AgNPs were in nanocrystal form and crystalline in nature. The peaks can be assigned to the planes (111), (200), (220), and (311) facet of silver crystal, respectively. For AuNPs, the characteristic diffraction peaks at 2θ values were predicted at 37.55, 44.73, 64.87 and 77.64, respectively. When compared with the standard (JCPDS card number 04‐0784), the obtained XRD spectrum confirmed that the mycosynthesized AuNPs were in nanocrystal form and crystalline in nature. The peaks can be assigned to the planes (111), (200), (220), and (311) facet of gold crystal, respectively. For CuNPs, the characteristic diffraction peaks at 2θ values were predicted at 32.69, 35.71, 38.51, 48.75, 53.70, 58.44, 61.68, 66.20, 68.14, 72.56 and 75.25, respectively. When compared with the standard (JCPDS card number 04‐0836), the obtained XRD spectrum confirmed that the mycosynthesized CuNPs were in nanocrystal form and crystalline in nature. The peaks can be assigned to the planes (110), (002), (111), (202), (020), (202), (113), (311), (113), (311) and (004) facet of Copper crystal, respectively. For ZnONPs, the characteristic diffraction peaks at 2θ values were predicted at 31.81, 34.48, 36.25, 47.56, 56.63, 62.95, 66.24, 67.94, 69.16 and 72.32, respectively. When compared with the standard (JCPDS card number 36‐1451), the obtained XRD spectrum confirmed that the mycosynthesized ZnONPs were in nanocrystal form and crystalline in nature. The peaks can be assigned to the planes (100), (002), (101), (102), (110), (103), (200), (112), (201) and (202) facet of Zinc oxide crystal, respectively. These XRD properties correspond to the reported properties of nanoparticles synthesized by a variety of fungi [4, 18, 42, 43].

#### 3.2.5 FTIR spectroscopy analysis

**FIGURE 6:**
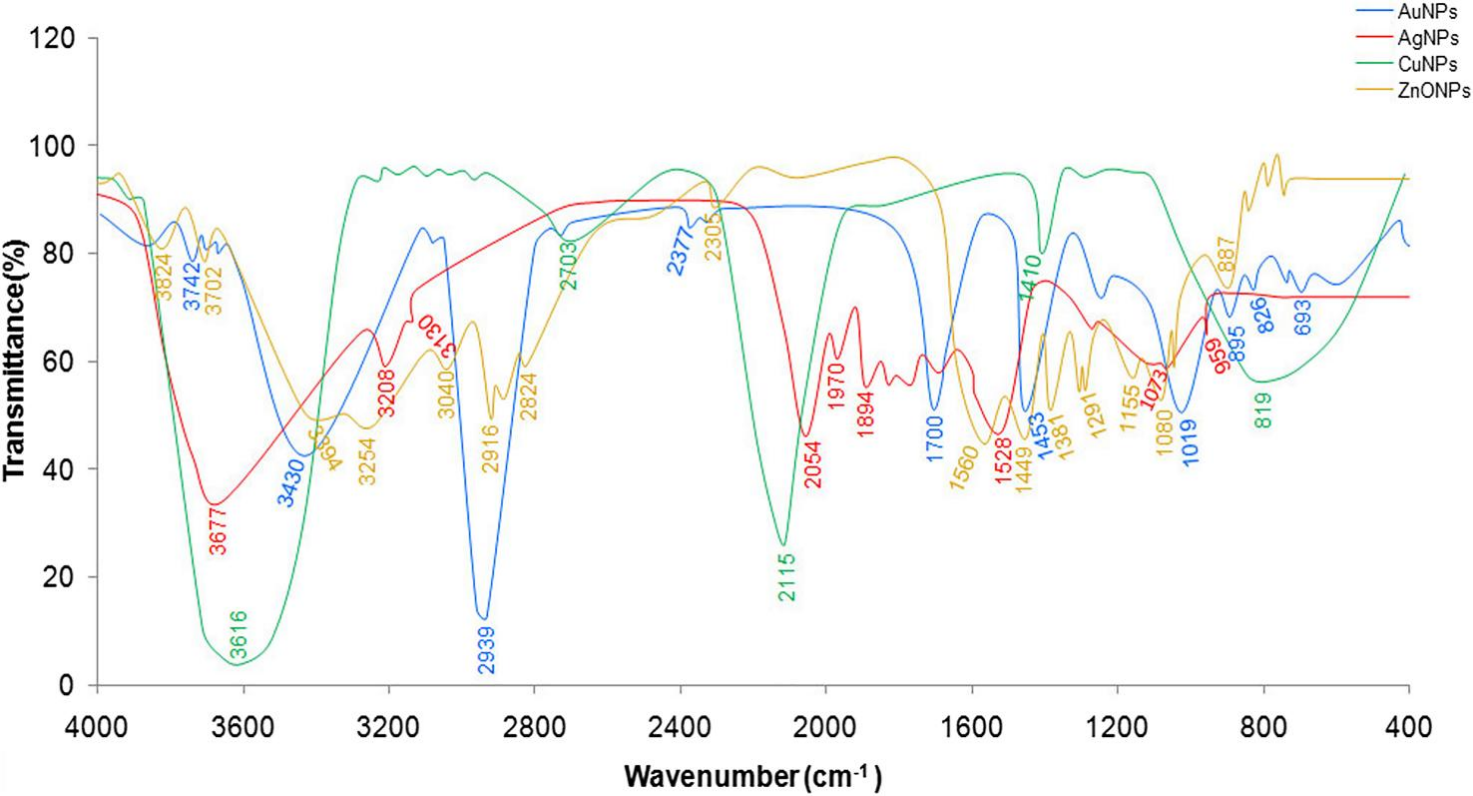
Fourier transform infrared spectroscopy (FTIR) spectra of mycosynthesized manufacture metallic nanoparticles (MNPs) (Ag, Au, Cu and ZnO) using the cell‐free filtrate of *Aspergillus kambarensis*. FTIR shows that proteins, amides, long chain fatty acids and polysaccharides can be considered as biological molecules that act as capping and stabilizing agents of MNPs along with bio‐reduction of metal ions

FTIR spectroscopy analysis indicated an interaction of the metal ions with the fungi cell‐free molecules secreted into water; which could be involved in bio‐reduction of metal ions, nucleation and/or stabilization of MNPs, as earlier has been reported by other fungal species [5, 6, 18]. The MNPs mycosynthesized by the fungal cell‐free filtrate of *A. kambarensis* were not in direct contact, signifying stabilization of the MNPs by a capping agent which was confirmed by FTIR analysis (Figure 6). The FTIR spectrum of mycobiosynthesized AgNPs using *A. kambarensis* culture filtrate, showed distinct peaks at 3677 (N–H stretching, primary amine), 3208 (O–H stretching, alcohol), 3130 (O–H stretching, alcohol), 2054 (C≡C stretching, alkyne), 1970 (C–H bending, aromatic compound), 1894 (C–H bending, aromatic compound), 1528 (C–H bending, alkane), 1073 (C–N stretching, amine) and 959 (C=C bending, alkene) cm^−1^. The FTIR spectrum of mycobiosynthesized AuNPs showed distinct peaks at 3742 (O–H stretching, alcohol), 3430 (N–H stretching, primary amine), 2939 (O–H stretching, carboxylic acid), 2377 (S–H stretching, thiol), 1700 (C=O stretching, primary amide), 1453 (C–H bending, alkane), 1019 (C–O stretching, primary alcohol), 895 (C=C bending, alkene), 826 (C=C bending, alkene), and 693 (C=C bending, alkene) cm^−1^. The FTIR spectrum of mycobiosynthesized CuNPs showed distinct peaks at 3616 (O–H stretching, alcohol), 2703 (O–H stretching, alcohol), 2115 (N=C=N stretching, carbodiimide), 1410 (O–H bending, carboxylic acid) and 819 (C=C bending, alkene) cm^−1^. The FTIR spectrum of mycobiosynthesized ZnONPs showed distinct peaks at 3824 (O–H stretching, alcohol), 3702 (O–H stretching, alcohol), 3394 (N–H stretching, primary amine), 3254 (O–H stretching, carboxylic acid), 3040 (C–H stretching, alkene), 2916 (C–H stretching, alkane), 2824 (C–H stretching, aldehyde), 2305 (S–H stretching, thiol), 1560 (C–H stretching, alkane), 1449 (O–H stretching, carboxylic acid), 1381 (O–H bending, alcohol), 1291 (C–N stretching, amine), 1155 (C–O stretching, ester), 1080 (C–O stretching, primary alcohol) and 887 (C=C bending, alkene) cm^−1^. The FTIR spectrum of mycogenic synthesized MNPs shows availability functional groups. So, proteins, amides, long chain fatty acids and polysaccharides can be considered as biological molecules that act as capping and stabilizing agents of MNPs along with bio‐reduction of metal ions.

### 3.3 Antimicrobial activity of MNPs

The results of disc diffusion technique for antimicrobial assay of biogenic Cu, Ag, Au and ZnO NPs were depicted in Table 1. All produced MNPs except AuNPs displayed antimicrobial properties against tested bacteria (inhibition zones of 18.0–44.1 mm) and fungi (inhibition zones of 13.4–33.0 mm). Overall, antibacterial activity of MNPs were higher than that of their antifungal activity. The activity of the Cu, Ag, Au and ZnO NPs against pathogenic fungi and bacteria has been reported by various researchers [44, 45, 46, 47, 48]. This activity has been shown to be influenced in part by several factors of synthesized nanoparticles such as size, surface modification, shape, surface charge, ligand type, concentration, and the culture medium and the source of synthesis [45]. AuNPs did not show any obvious antimicrobial activity in our study. There are several reports on unpredictable behaviour of AuNPs represented antibacterial activity [45], no antifungal activity but having antibacterial activity [46], no antifungal and antibacterial activity [47], and no antifungal activity [48]. In this relation, researchers showed that antibacterial activity of AuNPs completely depends on their effect on bacterial surface and subsequent alters the membrane potential [49] as well as the interaction between the bacterial membrane and the biomolecules from source of NP synthesis coating the AuNPs which may prevent the interaction of AuNPs with bacterial surface as a crucial step for inserting antibacterial activity via affinity towards the bacterial membrane [50]. Likewise, it has been shown that bactericidal activity of AuNPs reported in some studies may be related to co‐existing chemicals not completely removed from AuNPs [50]. So, no antimicrobial activity of synthesized AuNPs in the present study may be attributed to covering the bacterial and fungal surfaces by biomolecules of *A. kambarensis* during NPs synthesis. These AuNPs may be still beneficial as carriers or delivery vehicles of antibiotics/antifungals due to their non‐toxic effects on microbial pathogens and cancer cell lines.

**TABLE 1:** Antimicrobial activity of metallic nanoparticles against pathogenic bacteria and fungi by disc diffusion assay^a^

Microorganisms	Zone of inhibition (mm)									
	AgNPs (µg/ml)		CuNPs (µg/ml)		ZnONPs (µg/ml)		Streptomycin sulphate (µg/ml)		Fluconazole (µg/ml)	
	50	100	50	100	50	100	50	100	50	100
*Escherichia coli*	34.0 ± 1.0	42.1 ± 2.8	23.0 ± 2.6	37.1 ± 1.2	32.0 ± 1.1	44.1 ± 2.7	26.3 ± 1.5	34.2 ± 1.7	ND	ND
*Bacillus subtilis*	21.1 ± 1.5	29.4 ± 2.6	18.0 ± 1.2	27.1 ± 0.7	21.0 ± 0.5	30.5 ± 0.9	19.5 ± 1.7	30.2 ± 1.1	ND	ND
*Proteus vulgaris*	22.2 ± 0.5	32.1 ± 1.5	24.0 ± 0.7	30.2 ± 1.3	19.3 ± 0.8	31.5 ± 1.0	26.1 ± 0.8	44.2 ± 1.5	ND	ND
*Staphylococcus aureus*	27.1 ± 0.9	30.3 ± 1.7	26.1 ± 0.7	33.0 ± 1.0	18.1 ± 0.8	28.0 ± 0.8	24.0 ± 1.6	35.1 ± 1.6	ND	ND
*Candida albicans*	19.0 ± 0.6	25.1 ± 0.8	16.2 ± 1.2	21.0 ± 1.3	13.4 ± 1.0	20.1 ± 0.7	ND	ND	18.0 ± 2.9	27.2 ± 2.7
*Candida tropicalis*	26.1 ± 0.3	30.2 ± 1.3	23.2 ± 1.1	31.1 ± 0.9	16.2 ± 0.7	25.3 ± 0.9	ND	ND	25.0 ± 1.7	30.1 ± 1.8
*Fusarium oxysporum*	22.2 ± 1.6	31.0 ± 1.4	17.0 ± 1.0	24.1 ± 0.2	23.1 ± 1.2	33.0 ± 0.4	ND	ND	16.0 ± 0.5	26.2 ± 7
*Aspergillus niger*	25.1 ± 1.4	30.0 ± 0.9	15.1 ± 1.6	22.1 ± 1.1	22.0 ± 2.5	30.0 ± 2.5	ND	ND	17.1 ± 0.8	24.1 ± 0.7

Abbreviations: ND, not determined.

^a^ AuNPs did not show any obvious antimicrobial activity against tested fungi and bacteria.

### 3.4 In vitro cytotoxicity assay of MNPs

#### 3.4.1 MTT assay

**FIGURE 7:**
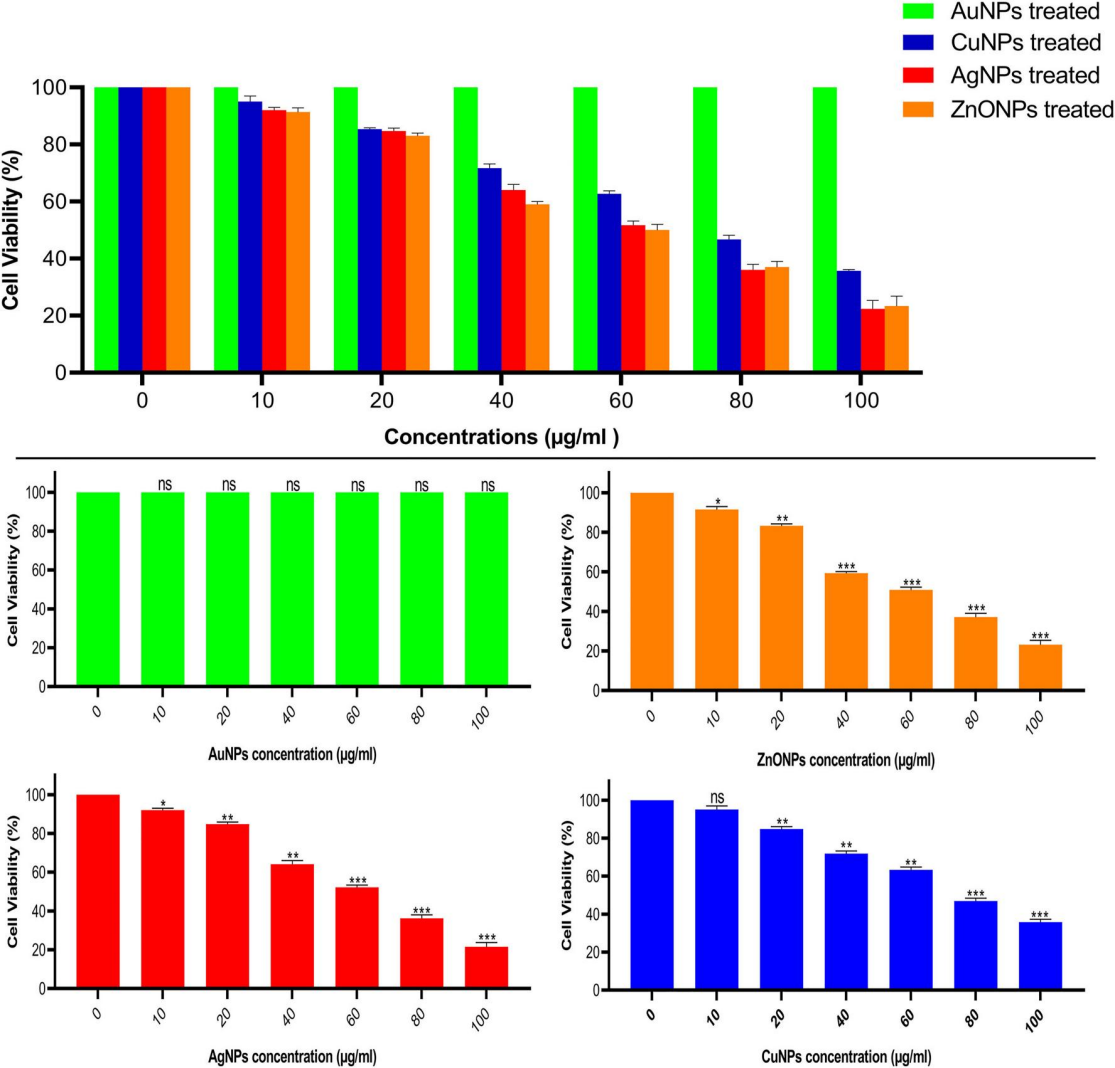
*In vitro* Cytotoxicity on HepG‐2 cells detection using MTT assay. Values are expressed as mean ± SD. All data were from three independent (*N* = 3) biological replicates in two separate experiments (*p ≤ 0.05, **p ≤ 0.01, ***p ≤ 0.001)

With the aim to estimate the cytotoxicity of mycosynthesized MNPs, in vitro cytotoxicity evaluates were accomplished with human liver cancer cell‐line (HepG‐2). Various concentrations of MNPs (0–100 µg/ml, 24 h) showed cytotoxicity properties on HepG‐2 cell‐lines. We found that the viability of cells declined with increasing concentrations of MNPs. Compared to the controls (sterile deionized water without MNPs), different concentrations of MNPs reduced the HepG‐2 viability as 21.55–92.05 for AgNPs, 35.81%–95.05% for CuNPs and 23.18%–91.47% for ZnONPs. AuNPs did not show any cytotoxic effect on HepG‐2 cell line (Figure 7). The IC_50_ of the Ag, Cu, and ZnO NPs for HepG‐2 were calculated after 24 h as 62.56, 77.03 and 62.01 µg/ml, respectively. The anticancer activity of the MNPs against human cancer cells has been reported in various studies [22, 23, 29, 32].

#### 3.4.2 Analysing cells by optical microscopy

**FIGURE 8:**
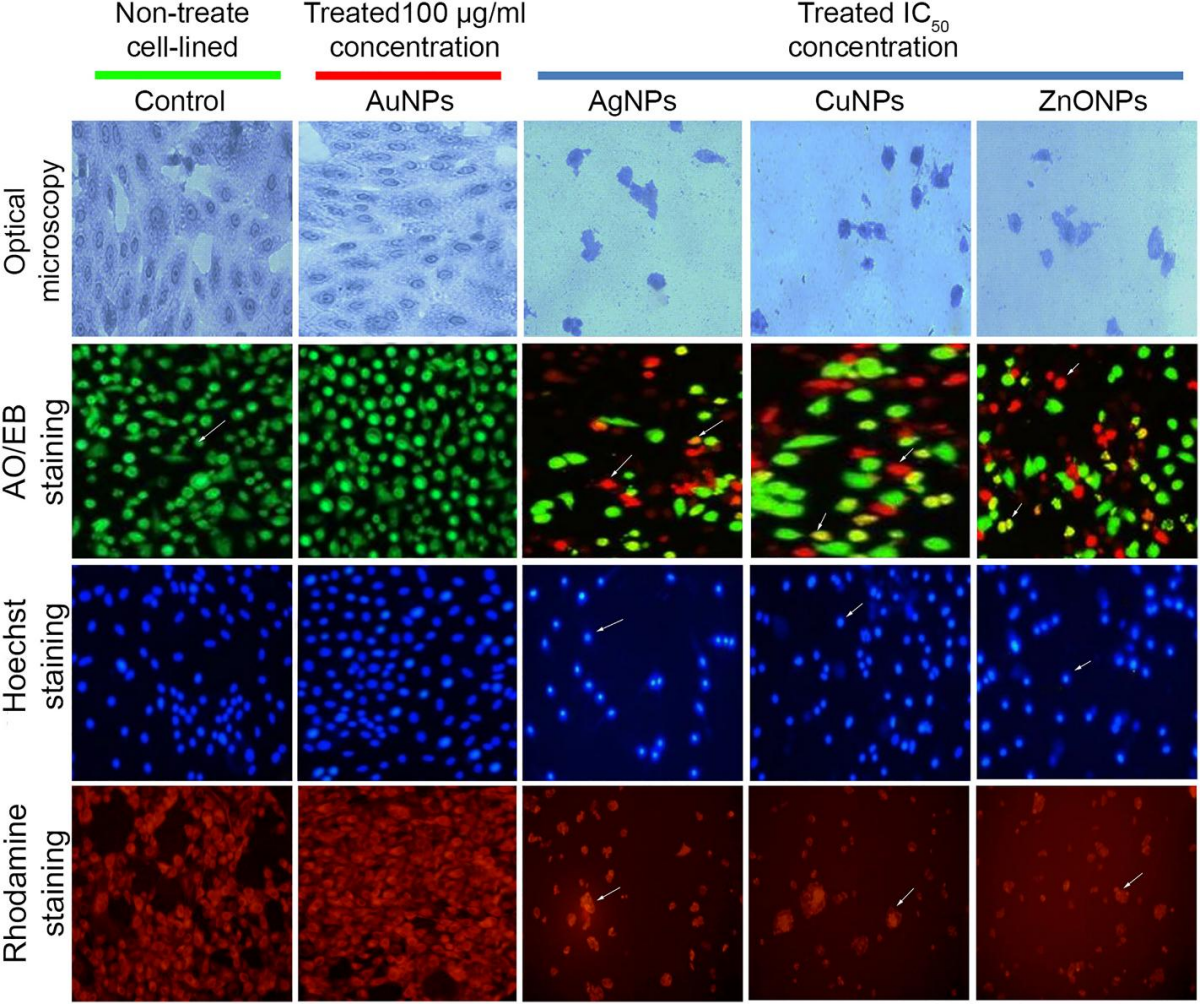
Optical and fluorescence microscopy images of mycogenic manufacture metallic nanoparticles (MNPs) treated using IC_50_ concentration of MNPs‐treated HepG‐2 cells and untreated control groups. AuNPs had no effect on cell viability similar to untreated control, while other MNPs showed various degrees of cell killing as evidenced in the figure. The scale bars are 20 µm

After treatment with mycogenic MNPs, the morphological changes and cellular morphologies were observed in treated HepG‐2 cell‐lines compared with normal/untreated cells. As shown in Figure 8, it was obvious that the chromatids of cancer cells were condensed, when treated with IC_50_ concentration of MNPs (Ag, Cu, and ZnO); as well as progressive structural modification and reduction of cancer cells populations were evident.

#### 3.4.3 Analysing cells by AO/EB

The induction of apoptosis plays a substantial role in drug discovery and development. Commercially accessible anticancer drugs have been exposed to induce apoptosis in cancer/susceptible cells. The morphology of the necrotic/dead, apoptotic and normal/untreated cells of HepG‐2 cells were recognized separately using fluorescence microscopy after staining by AO/EB based on the cell membrane integrity. HepG‐2 cells displayed green fluorescent colour that shows the presence of live cells in controls (untreated cells). Orange coloured cells contained pro‐apoptotic bodies and red necrotic/dead cells were found in the MNP‐treated HepG‐2 cell‐lines (Figure 8). Microscopic observation indicated that the untreated viable cells were detected to be green because of the binding of AO into cell membranes, whereas, pro‐apoptotic cells were detected as orange coloured bodies due to the shrinkage of nuclei and nuclear blebbing because of the induction of EB into cells. However, the dead/necrotic cells were turned into red colour due to their loss of outer membrane integrity by mycosynthesis MNPs as indicated by arrows (Figure 8).

#### 3.4.4 Analysing cells by Hoechst

Hoechst 33342 staining of normal HepG‐2 cancer cells (untreated) revealed regular morphology and an intact round nucleus which exhibited a weak blue fluorescence, whereas, MNP‐treated cells exhibited bright blue colour emission resulting in nuclear fragmentation by increased condensation of chromatin and leading to apoptosis in cancer liver cells (Figure 8). As exhibited in Figure 8, the numbers of apoptotic cells were increased when treated with MNPs (Ag, Cu, and ZnO). Nuclear morphology analysis displayed changes in apoptotic characters in the MNP‐treated HepG‐2 cells.

#### 3.4.5 Analysing cells by Rhodamine

Rhodamine 123 staining confirmed that the MNP‐treated HepG‐2 cells exhibited bright red colour emission resulting in mitochondrial fragmentation that led to initiation of apoptosis (Figure 8) while the normal/untreated cells had intact mitochondria and normal morphology that emitted a weak red fluorescence for HepG‐2 cells.

#### 3.4.6 Analysing cells by DNA fragmentation

**FIGURE 9:**
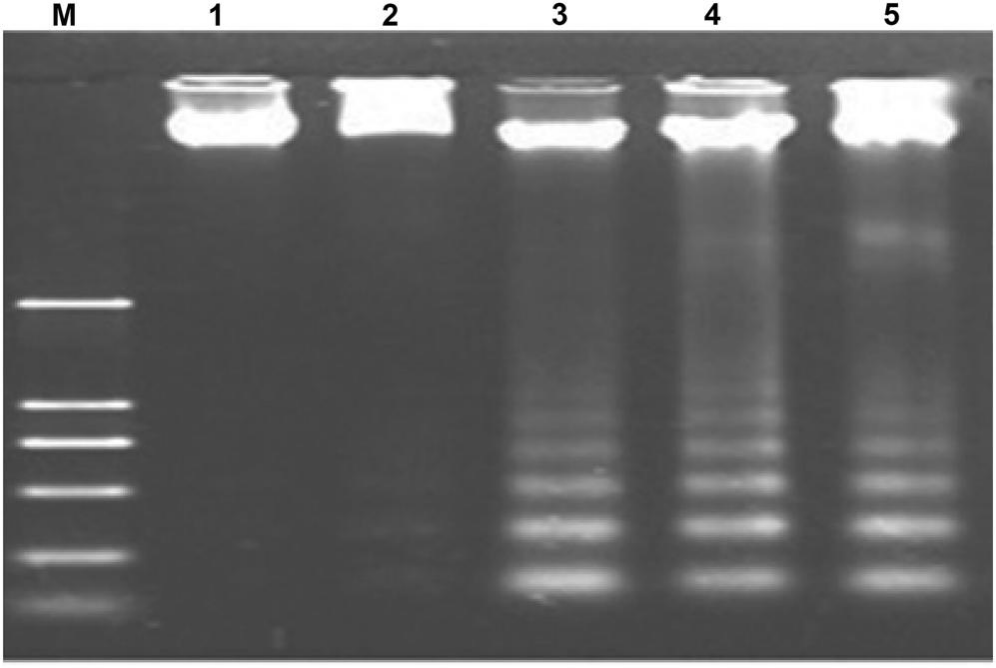
DNA fragmentation analysis of HepG‐2 cell‐lines exposed to manufacture metallic nanoparticles (MNPs). Lanes: M: 100 bp DNA ladder. 1: DNA from untreated HepG‐2 cells shows normal uniform band, 2, 3, 4, and 5: DNA from MNP‐treated HepG‐2 cells for AuNPs, AgNPs, CuNPs and ZnONPs, respectively. No fragmentation is evident for AuNPs indicating its lack of effectiveness, while massive fragmentation is seen for AgNPs‐, CuNPs and ZnONPs‐treated cells

Study of DNA fragmentation/damage was carried out to detect the death of HepG‐2 cells exposed to mycosynthesized MNPs. Agarose gel electrophoresis was used for observing the DNA fragmentation. DNA samples obtained from MNP‐treated cell‐lines along with untreated control were loaded on agarose gel. The results indicated that DNA fragmentation was detected in the MNP‐treated sample, while intact DNA band was noticed in the control (normal/untreated cells). Therefore, the HepG‐2 cell‐line was considered to be sensitive to biogenic MNPs at the IC_50_ dose. So, it appears that inhibitory effects of MNPs may be related in part to DNA fragmentation/damage mechanisms. HepG‐2 cells exposed to AuNPs did not show any obvious DNA fragmentation/damage (Figure 9).

## 4 CONCLUSIONS

In the present study, MNPs of Ag, Au, Cu and ZnO were successfully green synthesized by using the cell‐free filtrate of *A. kambarensis* as a novel source of nanoparticle synthesis. Extracellularly produced MNPs are cheap, favour large‐scale production due to cell‐free preparation, do not require additional steps such as ultrasound, chemicals or reaction with detergents to remove undesirable components and need simple downstream process for purification. So, these MNPs may be suitable in biomedical applications as potential therapeutic agents in antimicrobial and anticancer investigations due to their strong biological activities against human pathogenic fungi and bacteria and killing effects towards cancer HepG‐2 cell line. Further studies on in vivo cytotoxicity and antimicrobial activity of synthesized MNPs using animal experimental models are recommended.

## CONFLICT OF INTEREST

The authors declare that they have no competing interest.

## PERMISSION TO REPRODUCE MATERIALS FROM OTHER SOURCES

None.

## Data Availability

The data that support the findings of this study are available from the corresponding author upon reasonable request.
